# EVENT-SPECIFIC EMOTIONAL EXPRESSION OF PERSONS WITH DEMENTIA IN LONG-TERM CARE: PRELIMINARY RESULTS

**DOI:** 10.1093/geroni/igz038.1570

**Published:** 2019-11-08

**Authors:** Kyung Hee Lee, Ji Yeon Lee, Bora Kim, Marie Boltz

**Affiliations:** 1 Yonsei University College of Nursing, Seoul, Korea, Republic of; 2 Yonsei University, Seoul, Korea, Republic of; 3 Pennsylvania State University, University Park, Pennsylvania, United States

## Abstract

Although dementia-related language, comprehension, and memory deficits occur fairly early stage in dementia, persons with dementia retain the ability to express their emotion even in the late stage of disease. However, health care providers do not know how to interpret emotional expressions that could be utilized as important signals of underlying needs and care preferences in persons with dementia. The purpose of this study was to explore the event-specific emotional expressions of persons with dementia in long-term care over a 6-month period. This was a longitudinal study using repeated observations. Emotional expressions were videotaped when three specific events (personal care, meal time, and activity) occurred at baseline, month 3 and month 6. A total of nine observations was made for each participant. We enrolled 13 participants so far; ten participants were completed 6 month follow up. The mean score of MMSE at baseline was 4.38; that of ADL was 16.62. On average, persons with dementia showed 9.93 episodes of positive emotional expression (PEE) per minute and 1.81 episodes of negative emotional expression (NEE) per minute. We found between person variations for both PEE and NEE. PEE and NEE were different by three types of events. Specifically, persons with dementia showed more PEE with activity than personal care and meal time and more NEE with personal care than the other two events. This study will provide better understanding of event-specific emotional expressions, and inform the development of emotion-oriented interventions programs to improve the psychological well-being of persons with dementia.

